# Correction: Leung et al. Preclinical Identification of Sulfasalazine’s Therapeutic Potential for Suppressing Colorectal Cancer Stemness and Metastasis through Targeting KRAS/MMP7/CD44 Signaling. *Biomedicines* 2022, *10*, 377

**DOI:** 10.3390/biomedicines13030547

**Published:** 2025-02-21

**Authors:** Wai-Hung Leung, Jing-Wen Shih, Jian-Syun Chen, Ntlotlang Mokgautsi, Po-Li Wei, Yan-Jiun Huang

**Affiliations:** 1 Division of Colon and Rectal Surgery, Department of Surgery, Mackay Memorial Hospital, No. 92, Sec. 2, Zhongshan N. Rd., Taipei 10449, Taiwan; leungwh22@gmail.com (W.-H.L.); b101091039@tmu.edu.tw (J.-S.C.); 2 Ph.D. Program for Cancer Molecular Biology and Drug Discovery, College of Medical Science and Technology, Taipei Medical University and Academia Sinica, Taipei 11031, Taiwan; shihjw@tmu.edu.tw (J.-W.S.); d621108006@tmu.edu.tw (N.M.); 3 Graduate Institute of Cancer Biology and Drug Discovery, College of Medical Science and Technology, Taipei Medical University, Taipei 11031, Taiwan; 4 TMU Research Center of Cancer Translational Medicine, Taipei Medical University, Taipei 11031, Taiwan; 5 Ph.D. Program for Translational Medicine, College of Medical Science and Technology, Taipei Medical University, Taipei 11031, Taiwan; 6 Division of Colorectal Surgery, Department of Surgery, Taipei Medical University Hospital, Taipei Medical University, Taipei 110, Taiwan; poliwei@tmu.edu.tw; 7 Department of Surgery, School of Medicine, College of Medicine, Taipei Medical University, Taipei 110, Taiwan; 8 Division of General Surgery, Department of Surgery, Taipei Medical University Hospital, Taipei Medical University, Taipei 110, Taiwan

## Error in Figure

In the original publication, there is a mistake in Figure 11B as published [1]. The images of the colony formation assay are misplaced. The corrected Figure 11 appears below. The authors state that the scientific conclusions are unaffected. This correction was approved by the Academic Editor. The original publication has also been updated.

## Figures and Tables

**Figure 11:**
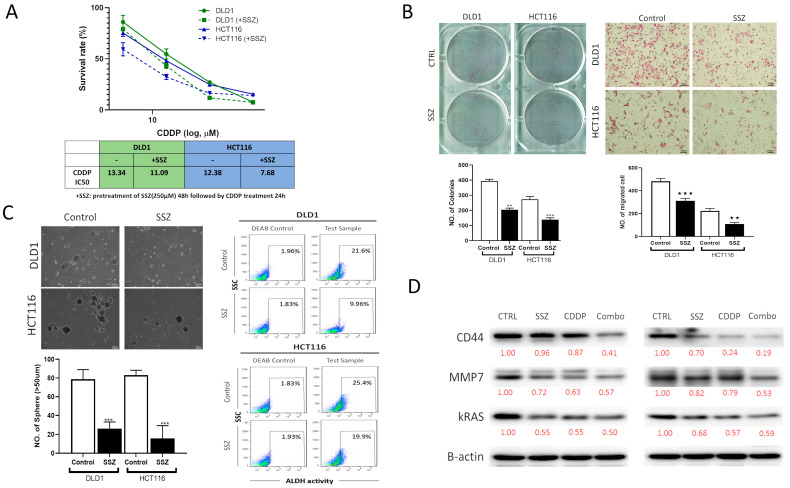
Sulfasalazine (SSZ) treatment reduced the tumorigenic properties of CRC cells and enhanced cisplatin (CDDP) efficacy. (**A**) SSZ enhanced cisplatin efficacy in both the DLD-1 and HCT116 cell lines. IC50 values are shown. Representative micrographs for the suppressive effects of SSZ on the ability of DLD-1 and HCT116 cells to form (**B**) colonies and migration, and (**C**) tumorspheres, as well as showing reduced ALDH activity (cancer stemness marker) in DLD-1 and HCT116 cells. (**D**) Western blot results show that SSZ and CDDP combined treatment significantly reduced the expression level of KRAS, MMP7, and CD44 on CRC cells compared to their vehicle-treated counterparts. β-actin served as the loading control. ** *p* < 0.01, *** *p* < 0.001. Numbers in red represent the relative expression level of the band intensity estimated using ImageJ software.
